# Fluorescence Lifetime Imaging of Quantum Dot Labeled DNA Microarrays

**DOI:** 10.3390/ijms10041930

**Published:** 2009-04-24

**Authors:** Gerard Giraud, Holger Schulze, Till T. Bachmann, Colin J. Campbell, Andrew R., Mount, Peter Ghazal, Mizanur R. Khondoker, Alan J. Ross, Stuart W. J. Ember, Ilenia Ciani, Chaker Tlili, Anthony J. Walton, Jonathan G. Terry, Jason Crain

**Affiliations:** 1 School of Physics and Astronomy, The University of Edinburgh, The King’s Buildings, West Mains Road, Edinburgh EH9 3JZ, Scotland, UK; E-Mail: jason.crain@ed.ac.uk (J.C.); 2 Division of Pathway Medicine, Medical School, The University of Edinburgh, Chancellor’s Building, Little France Crescent, Edinburgh EH16 4SB, Scotland, UK; E-Mails: holger.schulze@ed.ac.uk (H.S.); till.bachmann@ed.ac.uk (T.T.B.); colin.campbell@ed.ac.uk (C.J.C.); pghazal@uun.ed.ac.uk (P.G.); mkhondok@uun.ed.ac.uk (M.R.K.); aross@uun.ed.ac.uk (A.J.R.); sember@uun.ed.ac.uk (S.W.J.E.);; 3 School of Chemistry, The University of Edinburgh, Joseph Black Building, West Mains Road, Edinburgh, EH9 3JJ, Scotland, UK; E-Mails: amount@uun.ed.ac.uk (A.R.M.); iciani@staffmail.ed.ac.uk (I.C.); chaker_tlili@yahoo.fr (C.T.);; 4 Institute of Integrated Systems, School of Engineering, The University of Edinburgh, The King’s Buildings, West Mains Road, Edinburgh EH9 3JF, Scotland, UK; E-Mails: anthony.walton@ed.ac.uk (A.J.W.); jon.terry@ed.ac.uk (J.G.T.); 5 National Physical Laboratory, Hampton Road, Teddington, Middlesex TW11 0LW, UK

**Keywords:** Quantum Dots (QD), Fluorescence Lifetime Imaging Microscopy (FLIM), DNA Microarray, Lifetime Multiplexing

## Abstract

Quantum dot (QD) labeling combined with fluorescence lifetime imaging microscopy is proposed as a powerful transduction technique for the detection of DNA hybridization events. Fluorescence lifetime analysis of DNA microarray spots of hybridized QD labeled target indicated a characteristic lifetime value of 18.8 ns, compared to 13.3 ns obtained for spots of free QD solution, revealing that QD labels are sensitive to the spot microenvironment. Additionally, time gated detection was shown to improve the microarray image contrast ratio by 1.8, achieving femtomolar target sensitivity. Finally, lifetime multiplexing based on Qdot525 and Alexa430 was demonstrated using a single excitation-detection readout channel.

## 1. Introduction

DNA and protein microarrays are important tools for biomolecular detection with applications ranging from gene expression to clinical diagnostics [1–3]. In recent years microarray technology has benefited from advances in microfluidic devices, allowing the technique to be integrated in μ-TAS and point of care devices [4]. In general terms, microarray technology exploits molecular recognition between a probe molecule attached to a substrate and a complementary target, which may be a nucleic acid or protein. One of the major advantages of the technique lies in its unique multiplexing capabilities that allow thousands of probe spots to be printed on a single substrate. The read out of microarrays can be achieved using a range of detection techniques [5,6], however, fluorescence is the most commonly used transduction method. Typically, a cyanine dye is covalently attached to the target molecule. After incubation of the sample with the microarray, the emission intensity of the dye is used to determine whether selective binding of the target to the immobilized probe occurs. A number of microarray experiments have benefited from the multiple labeling of separate test samples, therefore permitting detection of differential expression without the need for additional arrays [7,8]. For example, this strategy has been employed for expression profiling microarrays [9], in which the expression of several genes in two samples of the same biological specimen (e.g. diseased and healthy tissue), are compared. Typically, DNA prepared from the two samples are labeled with two dyes with distinct emission bands, such as Cy3 (570 nm) and Cy5 (670 nm). The two labeled samples are then mixed and hybridized to a single microarray. However, fluorescence intensity detection methods suffer a number of disadvantages. Sensitivity is often restricted by the background fluorescence emitted from the microarray substrate and any chemical impurities present. The multiplexing of labels is also limited by the broad emission profiles associated with most organic dyes and requires a complex experimental setup, incorporating several excitation wavelength and detection channels.

In this manuscript, we explore an alternative transduction approach that combines quantum dot (QD) labeling and fluorescence lifetime imaging microscopy [10]. Quantum dots possess a number of advantages over traditional dyes, such as: a high quantum yield, long photostability, potential for multiplexing and a relatively long excited state lifetime [11]. These properties have triggered a strong interest within the biosensing community [12–16] resulting in QD being used to label antigen and oligonucleotide microarrays [17–19]. At the same time, the reading of DNA microarrays using time-resolved fluorescence techniques has been suggested as a potential improvement to the sensitivity of assay detection by filtering the background fluorescence of the substrate, using both cyanine and infrared dyes [20,21]. The first part of this paper presents data on fundamental lifetime characteristics of QD and QD labeled DNA spots. In the second part, we explore potential advantages and future applications of QD labeling combined with fluorescence lifetime detection, both in terms of sensitivity and multiplexing capabilities.

## 2. Experimental Section

### 2.1. Microarray production and incubation

DNA microarray assays performed in this study can be divided into three stages: 1.) the manufacture of the DNA microarray, 2.) the target hybridization, and 3.) the incubation of the QD labels. Human cytomegalovirus (HCMV) [22] and hepatitis C virus (HCV) oligonucleotides [23] (listed Table 1), were obtained from Eurogentec (Seraing, Belgium) and Metabion (Martinsried, Germany) respectively. Quantum dots (Qdot605 and Qdot525) functionalized with streptavidin were purchased from Invitrogen (Paisley, UK).

All DNA microarrays were prepared on epoxy silane coated glass slides (Nexterion slide E) obtained from Schott (Jena, Germany). First, thiol-modified probes were spotted at a concentration of 20 μM in Schott Nexterion spotting buffer containing 5 mM of tris(2-carboxyethyl) phosphine hydrochloride (TCEP) using a Microgrid II (Genomic Solutions, Cambridge, UK) with 200 μm solid pins and immobilized by 60 min incubation in a humidification box at room temperature, followed by incubation over night at room temperature in a closed box. Non-covalently bound probes were then removed using the Schott wash protocol consisting of four separate washing steps: 5 min in 0.1% Triton X100 at room temperature (RT), 4 min in 1 mM HCl solution at RT, 10 min in 100 mM KCl and 1 min in deionised water. The remaining free epoxy groups were blocked by immersion of the slide for 15 min in a solution of 50 mM ethanolamine in 100 mM Tris HCl buffer at pH 9.0 at 50 ºC, followed by a 1 min wash in water. The slides were dried by centrifugation at 1,200 g for 3 min.

Hybridization of biotinylated DNA target was performed with a 25 μL Gene Frame (Thermo Scientific, Surrey, UK) in a solution of 4x sodium chloride sodium citrate (SSC; 600 mM NaCl + 60 mM Na-citrate) buffer and 0.01% sodium dodecyl sulphate (SDS) for two hours at 55 ºC and 1400 rpm with an Eppendorf thermomixer (Eppendorf, Hamburg, Germany). Unspecifically bound target DNA was removed by washing the slide for 10 min in 2x SSC + 0.2% SDS followed by 10 min in 2x SSC and finally 10 min in 0.2x SSC. Slides were dried by centrifugation at 1,200 g for 3 min after a short immersion in deionised water.

Hybridized arrays were incubated with 20 nM Qdot605-streptavidin-conjugate solution (Invitrogen, Paisley, UK) in Invitrogen QD incubation buffer (50 mM borate with 2% BSA, pH 8.3) for one hour at 30 ºC and 1,400 rpm with an Eppendorf thermomixer. A final washing step of 10 min in 1x SSC + 0.1% SDS followed by 10 min in 0.2x SSC was required in order to remove unbound labels. The slides were then dried by centrifugation at 1,200 g for 3 min.

### 2.2. TIRF-FLIM experimental setup

**Figure 1.**
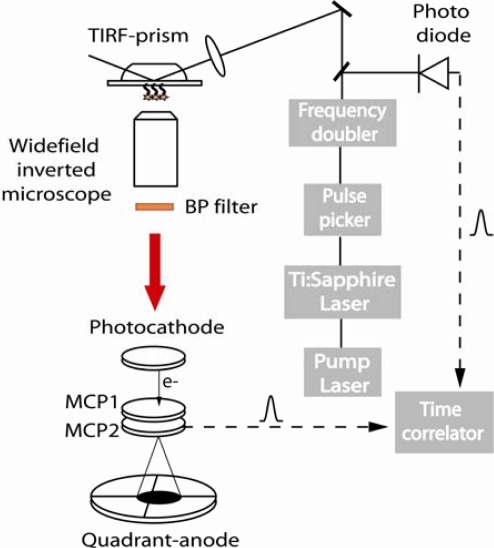
Experimental setup of the fluorescence lifetime imaging system based on TIRF excitation and Quadrant Anode detection. (See text for details).

A schematic of the experimental setup is shown in Figure 1. The excitation source was a femtosecond Ti:Sapphire laser system (10W Verdi and Mira from Coherent, Glasgow, Scotland) producing pulses of 200 fs at 76 MHz. The output of the Mira was passed through a pulse picker, reducing the repetition rate to 826 kHz, and then frequency doubled to give an output at 405 nm. The resultant excitation beam was subsequently split into two channels. A fraction of the excitation beam was sent onto a fast photodiode connected to a constant fraction discriminator which delivered the trigger signals. The remaining portion of the excitation beam was directed to excite the DNA microarray by total internal reflection. In order to achieve this, a quartz prism (Cairn Research, Faversham, UK) was attached to the condenser of a Nikon TE300 inverted microscope and placed into contact with undersurface of the DNA microarray using transmission immersion oil. The laser beam was directed below critical angle and focused on to the DNA microarray, generating a local evanescent excitation of circa 1 mm^2^ area. The resulting fluorescence was then collected with a plan apo X10 objective, filtered with an emission band pass filter (605/55 nm or 535/40 nm) and imaged onto a time- and space-correlated single photon counter detector (Quandrant Anode (QA) from EuroPhoton, Berlin, Germany) [24].

### 2.3. Image acquisition and analysis

Data acquisition was performed in reverse mode, with each detected photon assigned to one of 4,096 channels, each of width 27 ps. Typically, the count rate over the entire detector was 20 kHz, giving an acquisition time of ~ 15 min. During this time, an area of 1 mm^2^ containing up to 12 spots was imaged with a resolution of 10,000 pixels. Data analysis was performed by selecting an area of interest of the image and by extracting its emission decay curve. Estimate of fluorescence lifetime was obtained via a tail-fitting procedure from the peak of the decay using F900 software (Edinburgh Instruments, Livingston, UK). Counts in the peak channel were typically around 10^4^. Quality of the fit was judged on the basis of the reduced chi-squared statistic, χ^2^, and the randomness of residuals. Lifetime maps were generated using QA Analysis software (EuroPhoton, Berlin, Germany) and background noise estimated by taking the mean counts per pixel (cpp) of the background and subtracting this level from the entire image. A lifetime map was then produced by assigning a color on a 16-bit pseudocolor scale to a fitted single exponential decay time, and these were displayed over a range of 0 – 20/30 ns.

## 3. Results and Discussion

### 3.1. Fluorescence Lifetime Imaging of Quantum Dots and Quantum Dot-Labeled DNA Microarray spots

**Figure 2.**
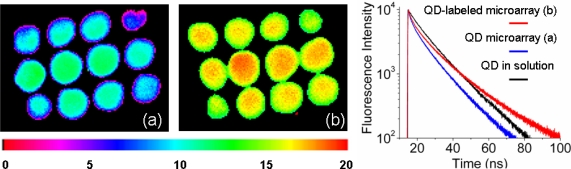
(Left) FLIM images of (a) QD microarray (1 nM) and (b) HCMV DNA microarray hybridized with 1 nM HCMV-QD labeled target. (Right) time- resolved fluorescence decays extracted from (b) red curve and (a) blue curve. For comparison the fluorescence lifetime of Qdot605 measured in solution is shown in black. Fitting parameters are listed in Table 2.

The experiments in this section were performed using two types of array: 1.) “QD microarrays” were constructed by spotting a matrix of twelve solubilized Qdot605-streptavidin-conjugates (1nM) on to epoxy silane coated glass slides and 2.) “QD-labeled DNA microarrays” were constructed by spotting twelve HCMV DNA probes on to epoxy coated glass slides and subsequently hybridizing with biotinylated complementary HCMV target labeled with Qdot605-streptavidin-conjugates. Figure 2 shows the FLIM images obtained for these two types of microarray set-ups. The FLIM map of the “QD-labeled DNA microarray” in Figure 2 (b) shows a distinctly longer lifetime than the “QD microarray” in Figure 2 (a). The lifetimes extracted from the spots are displayed in Figure 2 (right) and their fit reported in Table 2. All curves display multiexponential emission decays.

The complex emission of QD has been reported previously both for ensemble and single molecule measurements [25,26]. Such multiple components are typically associated with the presence of surface trap states that lie within the band gap of the nanocrystal and provide alternative pathways of the excited state relaxation. Here, we fit the data in Figure 2 with a sum of three exponential decays, a short (~ 2 ns), medium (10 – 12 ns), and long component (22 – 30 ns), to give quantitative values for the time scales involved.

Noticeably, the multiexponential behavior is more pronounced when measuring QD spots (blue curve), compared to the QD solution (black curve). This is also confirmed by the poorer χ^2^ value obtained on the glass substrate. The long component of the QD spot is clearly shorter with a value of 22 ns compared to 27 ns. This effect is thought to be due to local inhomogeneity following possible clustering effects. More importantly, QD and QD-DNA spots show substantially different emission decays. We observe that spots on the “QD microarray” (blue curve) had a distinctively shorter lifetime compared to spots on the “QD-labeled DNA microarray” (red curve). In the latter case, the spots displayed a longer third component with a value of 30 ns compared to only 22 ns. This resulted in an overall characteristic lifetime T_c_ of 18.8 ns, almost 6 ns longer than the T_c_ calculated for the QD spots. These differences suggest that QD are sensitive to their local environment, e.g. their interaction with DNA.

The quantum dots used in this study consisted of a CdSe core capped with ZnS. The core-shell material was further coated with a polymer functionalized with streptavidin, giving the nanocrystal water-soluble and biocompatible properties. Interference with either the passivation or functionalized layer can lead to changes in the excited state lifetime of the nanocrystal. For instance, capping the CdSe core with a ZnS passivation layer lengthens the lifetime of the QD. Since the ZnS passivation layer is not perfectly homogeneous, it has been suggested that surfactant molecules could complete this layer and thereby further lengthen the trap state and hence emission lifetime [27]. When varying the ligand coating layer, more dramatic variations in fluorescence lifetime has been observed [28,29]. The careful measurements of radiative and nonradiative decay rates of CdSe/Zns quantum dots capped with different thioalkyl acids, revealed that the luminescence of such nanocrystals is in fact dependant on a sum of complex factors. These include changes in dielectric constants surrounding the QD, interaction with the hole of the exciton wavefunction, and to a lesser extent passivation. In light of this study, one can speculate that similar interactions involving DNA and the functionalized layer of the QD are responsible for the long fluorescence lifetime observed on the QD-labeled DNA microarray.

### 3.2. Background reduction

In this section we investigated the benefit of using time-gated detection as a tool to enhance the image contrast of DNA microarrays. Similar approaches have been previously reported using organic dyes [20,21]. Here, we show that this technique can be applied at femtomolar target concentration. To do this, we hybridized a biotinylated complementary HCMV target labeled with Qdot605 streptavidin conjugates, to a HCMV DNA microarray.

**Figure 3.**
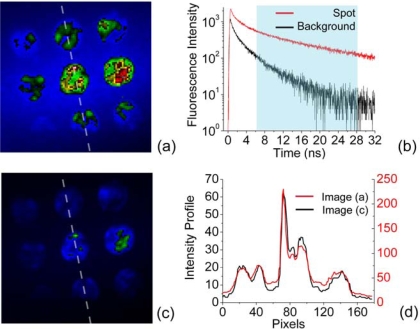
(a) Image intensity of DNA microarray incubated with 500 fM of QD-labeled HCMV target obtained by integrating all 4000 channels. (b) Fluorescence decays extracted from the spot (red) and the surrounded background (black). (c) Image intensity obtained by selecting only a time window between 6 and 28 ns. (d) Intensity profiles measured across three spots (dash line), extracted from image (a) (red) and image (c) (black).

Data acquisition using Time Correlation Single Photon Counting (TCSPC), produces histograms of photon arrival times for each pixel in the image. These histograms can then be manipulated and analyzed in various ways. Here, the image contrast was obtained by calculating the ratio of the signal over background value integrated over an area of 180 pixels for a succession of different time windows. A maximum contrast was obtained by starting the integration at 6.3 ns after the excitation pulse and stopping it after 28 ns [Figure 3(b)] leading to a gain in contrast of 1.8 fold.

The unprocessed image with all detected photons, is displayed in Figure 3(a) and is shown compared with an image covering the same respective spots constructed only from photons arriving in the time window between 6 and 28 ns after the laser pulse Figure 3(c). The intensity profile measured across three spots [Figure 3(d)] for the raw image (red) and gated image (black), shows a significant reduction in background noise.

### 3.3. Multiplexed Label Detection

The implementation of lifetime discrimination into multiple label microarray applications could greatly simplify the detection scheme by using a single readout channel. As a proof of concept experiment, we prepared a DNA microarray spotted with two different DNA probes and hybridized with their distinct complementary target conjugates. The microarray consisted of three sub-arrays comprising nine spots, incubated with a) HCV probe, b) HCMV probe and c) a 1:1 molar ratio of HCV and HCMV probe. The array was hybridized with a solution of complementary target containing 10 nM of Alexa Fluor 430 (Alexa430) labeled HCV target and 10 nM biotinylated HCMV target, which was further incubated with 20 nM Qdot525-streptavidin-conjugate solution. Qdot525 and Alexa430 were chosen for their overlapping absorption and emission spectra combined with a distinctly different excited state lifetime, approximately 4 and 25 ns, respectively [30,31]. Those properties permitted FLIM measurement without having to change the experimental apparatus by exciting the array at 405 nm and collecting the emission with a single band pass filter 535/40 nm.

**Figure 4.**
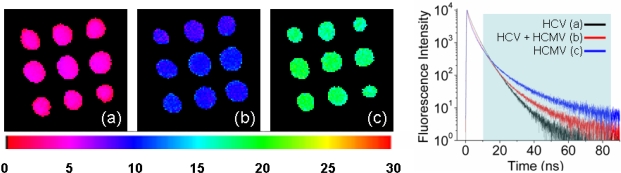
(Left) FLIM maps extracted from a series of three microarrays spotted with: (a) HCV probe, (b) probe spots containing 50% HCV and 50% HCMV and (c) HCMV probe. All three arrays were hybridized with a solution containing 10nM of Alexa430 labeled HCV complementary target and 10nM of Qdot525 labeled HCMV complementary target. (Right) Fluorescence lifetime traces extracted from (a)-black, (b)-red and (c)-blue.

The FLIM maps obtained for each of the three sub-arrays are presented in Figure 4 along with their respective emission decay traces. Each trace has been fitted with a maximum of four exponential decays as reported in Table 3. The decay extracted from the Alexa-labeled HCV spots are reasonably well described with a three exponentials fit leading to a characteristic lifetime of 4.2 ns, which is in agreement with the literature [31]. The fluorescence lifetimes extracted from the QD-labeled HCMV and the mixed HCV-HCMV array show a more complex behavior and a very poor fit. Even when using four decay components this is observed and is due to a relatively high contribution from the short lived fluorescence background. For this reason, the FLIM maps have been calculated within a time window spanning from 10 to 85 ns. The color coded lifetimes obtained give a good estimate of the label’s lifetime attached to the different microarrays: pink ~ 4 ns (Alexa), green ~ 20 ns (QD), and blue ~10 ns (mixture of Alexa and QD).

Quantum dots have a broad absorption band and are available with a wide range of emission characteristics; therefore they may be used in combination with a number of other dyes that present with a distinctly different lifetime. For instance, Lucifer Yellow with a lifetime of approximately 7 ns could be considered in order to further increase the dimension of the microarray.

## 4. Conclusions

We have successfully demonstrated alternative approaches to standard DNA microarray detection using a FLIM imager which combines evanescent wave excitation with wide field detection, using a quadrant anode mounted on an inverted microscope. This setup was used on a series of DNA microarrays for the detection of HCMV and HCV with quantum dot labeling. Fundamentally, fluorescence lifetimes from quantum dots spotted directly on to epoxy silane coated glass substrates were circa 6 ns shorter compared to quantum dot-labeled target DNA hybridized to capture probes. We anticipate a range of dielectric and electrostatic interactions such as the presence of DNA in the spot, to be responsible for this pronounced difference, and further studies will be required to precisely understand their full effects. We have demonstrated that this relatively long lifetime of quantum dots in a DNA spot can be used to increase the contrast ratio of DNA microarrays by a factor of 2, by time gating the signal acquisition. We have also shown that an appropriate set of labels with similar absorption and emission profiles, but distinct lifetimes, could be used efficiently in order to discriminate between various target DNAs. Our experiment demonstrates the capability of the technique to simplify multilabel microarray experiments by recording the data using a single channel for excitation and detection, whilst discriminating between different fluorescent lifetimes.

The technique is currently limited by a relatively long data acquisition time compared to conventional transduction method based on fluorescence intensity detection, which impair its use for high throughput screening applications. Such limitation could be overcome by the development of single-photon avalanche diodes (SPADs) arrays technology [32,33] for time resolved optical sensing, that allow TCSPC to be carried out in parallel for each detector. The approach demonstrated in our study in combination with technical advances made toward fast fluorescence lifetime detectors and compact picosecond laser systems, have a great potential toward commercially viable DNA and protein microarray solutions.

## Figures and Tables

**Table 1.** Synthetic HCV and HCMV oligonucleotides.

**HCMV target**	5′-AGTGTTGAGGGCCGTAAGCGTGTTGTGTCCGACGCTGCCTGCGCA CTGCCGGTGCGTGTCGTCCCACGGTATTTG – 3′ [5′] = Biotin-TEG
**HCMV probe**	5′-CAAATACCGTGGGACGACACGCACCGGCAGTGCGCAGGCAGCGT CGGACACAACACGCTTACGGCCCTCAACACT – 3′ [5′] = SH (C6)
**HCV target**	5′-GCGAAGGCTTGTGGTACTGCCTGATAGGGTGCTTGCGAGTGCCCC GGGAGGTCTCGTAGACCGTGCA [5′] = Alexa 430
**HCV probe**	5′-TGCACGGTCTACGAGACCTCCCGGGGCACTCGCAAGCACCCTATC AGGCAGTACCACAAGGCCTTTCGC - 3′ [5′] = SH

**Table 2.** Fluorescence lifetime measurement of Qdot605 samples. Each curve was fitted with a three component exponential-decay function; t_i_ are the lifetimes of each decay component and A_i_ their respective fractional intensity. χ^2^ is the reduced chi-squared statistic and T_c_ the characteristic lifetime calculated as: $T_{c}\;=\;\sum A_{i}t_{i}^{2}/\sum A_{i}t_{i}$.

Sample	Free QD Solution*	Free QD Glass	QD-dsDNA Glass
t1 (ns)	2.1 ± 0.05	1.6 ± 0.02	1.9 ± 0.02
t2 (ns)	12.1 ± 0.1	9.6 ± 0.05	11.1 ± 0.1
t3 (ns)	27.6 ± 0.3	21.8 ± 0.2	29.5 ± 0.2
A1 (%)	27.2 ± 0.4	32.4 ± 0.2	30 ± 0.2
A2 (%)	63.5 ± 0.3	54.1 ± 0.2	52.6 ± 0.2
A3 (%)	9.4 ± 0.4	13.4 ± 0.3	17.4 ± 0.3
χ^2^	1.3	1.5	1.4
Tc (ns)	15.3	13.3	18.8

* sample measured separately using a fluorometer from Edinburgh Instruments.

**Table 3.** Fluorescence lifetime extracted from three spots: HCMV probe labeled with QD-labeled complementary target, HCV probe labeled with Alexa-labeled complementary target, mixed spots of HCMV + HCV probes. Each curve was fitted with a three or four components exponential-decay function; t_i_ are the lifetimes of each decay component and A_i_ their respective fractional intensity. χ^2^ is the reduced chi-squared statistic and T_c_ the characteristic lifetime.

Spot	QD-labeled HCMV spot	Alexa-labeled HCV spot	Mixed spot HCMV/HCV
t1 (ns)	0.67 ± 0.01	1.4 ± 0.02	0.48 ± 0.05
t2 (ns)	2.5 ± 0.03	3.63 ± 0.03	2.1 ± 0.1
t3 (ns)	6.8 ± 0.1	7.5 ± 0.1	5 ± 0.3
t4 (ns)	23.9 ± 0.3	NA	16.8 ± 0.3
A1 (%)	36.1 ± 0.4	27.1 ± 0.7	20.6 ± 0.3
A2 (%)	48 ± 0.3	63.5 ± 0.5	47.2 ± 0.3
A3 (%)	14.7 ± 0.4	9.4 ± 0.5	31 ± 0.5
A4 (%)	1.25 ± 0.04	NA	1.25 ± 0.03
χ^2^	2.4	1.5	2.9
Tc (ns)	NA	4.2	NA

## References

[1] Petrik J (2006). Diagnostic applications of microarrays. Transfusion Med.

[2] Livingston AD, Campbell CJ, Wagner EK, Ghazal P (2005). Biochip sensors for the rapid and sensitive detection of viral disease. Genome Biol.

[3] Barl T, Dobrindt U, Yu XL, Katcoff DJ, Sompolinsky D, Bonacorsi S, Hacker J, Bachmann TT (2008). Genotyping DNA chip for the simultaneous assessment of antibiotic resistance and pathogenic potential of extraintestinal pathogenic Escherichia coli. Int. J. Antimicrob. Agents.

[4] Situma C, Hashimoto M, Soper SA (2006). Merging microfluidics with microarray-based bioassays. Biomol. Eng.

[5] Schaferling M, Nagl S (2006). Optical technologies for the read out and quality control of DNA and protein microarrays. Anal. Bioanal. Chem.

[6] Nagl S, Schaeferling M, Wolfbeis OS (2005). Fluorescence analysis in microarray technology. Microchim. Acta.

[7] Woo Y, Krueger W, Kaur A, Churchill G (2005). Experimental design for three-color and four-color gene expression microarrays. Bioinformatics.

[8] Lindroos K, Sigurdsson S, Johansson K, Ronnblom L, Syvanen AC (2002). Multiplex SNP genotyping in pooled DNA samples by a four-colour microarray system. Nucleic Acids Res.

[9] Shalon D, Smith SJ, Brown PO (1996). A DNA microarray system for analyzing complex DNA samples using two-color fluorescent probe hybridization. Genome Res.

[10] Suhling K, French PMW, Phillips D (2005). Time-resolved fluorescence microscopy. Photoch. Photobio. Sci.

[11] Resch-Genger U, Grabolle M, Cavaliere-Jaricot S, Nitschke R, Nann T (2008). Quantum dots versus organic dyes as fluorescent labels. Nat. Methods.

[12] Medintz IL, Uyeda HT, Goldman ER, Mattoussi H (2005). Quantum dot bioconjugates for imaging, labelling and sensing. Nat. Mater.

[13] Costa-Fernandez JM, Pereiro R, Sanz-Medel A (2006). The use of luminescent quantum dots for optical sensing. Trac-Trend Anal. Chem.

[14] Liu TC, Liu BS, Zhang HL, Wang Y (2005). The fluorescence bioassay platforms on quantum dots nanoparticles. J. Fluoresc.

[15] Sapsford KE, Pons T, Medintz IL, Mattoussi H (2006). Biosensing with luminescent semiconductor quantum dots. Sensors.

[16] Gill R, Zayats M, Willner I (2008). Semiconductor quantum dots for bioanalysis. Angew. Chem. Int. Edit.

[17] Kerman K, Endo T, Tsukamoto M, Chikae M, Takamura Y, Tamiya E (2007). Quantum dot-based immunosensor for the detection of prostate-specific antigen using fluorescence microscopy. Talanta.

[18] Shingyoji M, Gerion D, Pinkel D, Gray JW, Chen FQ (2005). Quantum dots-based reverse phase protein microarray. Talanta.

[19] Liang RQ, Li W, Li Y, Tan CY, Li JX, Jin YX, Ruan KC (2005). An oligonucleotide microarray for microRNA expression analysis based on labeling RNA with quantum dot and nanogold probe. Nucleic Acids Res.

[20] Valentini G, D’Andrea C, Comelli D, Pifferi A, Taroni P, Torricelli A, Cubeddu R, Battaglia C, Consolandi C, Salani G, Rossi-Bernardi L, De Bellis G (2000). Time-resolved DNA-microarray reading by an intensified CCD for ultimate sensitivity. Opt. Lett.

[21] Waddell E, Wang Y, Stryjewski W, McWhorter S, Henry AC, Evans D, McCarley RL, Soper SA (2000). High-resolution near-infrared imaging of DNA microarrays with time-resolved acquisition of fluorescence lifetimes. Anal. Chem.

[22] Chambers J, Angulo A, Amaratunga D, Guo HQ, Jiang Y, Wan JS, Bittner A, Frueh K, Jackson MR, Peterson PA, Erlander MG, Ghazal P (1999). DNA microarrays of the complex human cytomegalovirus genome: Profiling kinetic class with drug sensitivity of viral gene expression. J. Virol.

[23] Ember SWJ Ross AJ Schulze H Luby J Bachmann TT Crain J Walton AJ Ciani I Giraud G Mount AR Tlili C Terry JG Khondoker MR Ghazal P Campbell CJClinical applications of DNA and protein microarrays for the serodiagnosis of HCV infection To be submitted for publication.

[24] Emiliani V, Sanvitto D, Tramier M, Piolot T, Petrasek Z, Kemnitz K, Durieux C, Coppey-Moisan M (2003). Low-intensity two-dimensional imaging of fluorescence lifetimes in living cells. Appl. Phys. Lett.

[25] Grecco HE, Lidke KA, Heintzmann R, Lidke DS, Spagnuolo C, Martinez OE, Jares-Erijman EA, Jovin TM (2004). Ensemble and single particle photophysical proper-ties (Two-Photon excitation, anisotropy, FRET, lifetime, spectral conversion) of commercial quantum dots in solution and in live cells. Microsc. Res. Techniq.

[26] Fisher BR, Eisler HJ, Stott NE, Bawendi MG (2004). Emission intensity dependence and single-exponential behavior in single colloidal quantum dot fluorescence lifetimes. J. Phys. Chem. B.

[27] Jones M, Nedeljkovic J, Ellingson RJ, Nozik AJ, Rumbles G (2003). Photoenhancement of luminescence in colloidal CdSe quantum dot solutions. J. Phys. Chem. B.

[28] Kloepfer JA, Bradforth SE, Nadeau JL (2005). Photophysical properties of biologically compatible CdSe quantum dot structures. J. Phys. Chem. B.

[29] Algar WR, Krull UJ (2007). Luminescence and stability of aqueous thioalkyl acid capped CdSe/ZnS quantum dots correlated to ligand ionization. ChemPhysChem.

[30] Sutter JU, Macmillan AM, Birch DJS, Rolinski OJ, Wolfbeis OS (2008). Toward single-metal-ion sensing by Forster resonance energy transfer. Annals of the New York Academy of Sciences.

[31] Thomas AV, Herl L, Spoelgen R, Hiltunen M, Jones PB, Tanzi RE, Hyman BT, Berezovska O (2006). Interaction between presenilin 1 and ubiquilin 1 as detected by fluorescence lifetime imaging microscopy and a high-throughput fluorescent plate reader. J. Biol. Chem.

[32] Niclass C, Favi C, Kluter T, Gersbach M, Charbon E (2008). A 128 × 128 Single-Photon Image Sensor With Column-Level 10-Bit Time-to-Digital Converter Array. IEEE J. Solid-St. Circ.

[33] Charbon E (2008). Towards large scale CMOS single-photon detector arrays for lab-on-chip applications. J Phys D: Appl Phys.

